# Reconstructive hip surgery short-term outcomes and redislocation rate in non-ambulatory children with spastic cerebral palsy: a prospective cohort study

**DOI:** 10.1186/s12891-026-09852-4

**Published:** 2026-05-07

**Authors:** Ahmed S. Nagy, Mazen S. Aboulesaad, Ahmed M. Saied, Khaled Zaghloul

**Affiliations:** Department of Orthopaedic Surgery, Mansoura University Hospitals, Faculty of Medicine, Mansoura University, Mansoura, Dakahlia Egypt

**Keywords:** Cerebral palsy, Hip displacement, Varus derotational osteotomy, Dega pelvic osteotomy, CPCHILD

## Abstract

**Background:**

Hip displacement is a major cause of pain and reduced quality of life in non-ambulatory children with spastic cerebral palsy. Reconstructive hip surgery aims to restore stable femoral head containment, improve comfort, and facilitate daily care. This study evaluated short-term radiographic and caregiver-reported outcomes after reconstructive hip surgery in non-ambulatory children with spastic cerebral palsy, including exploratory subgroup analyses of varus derotational osteotomy (VDRO) alone and VDRO combined with Dega pelvic osteotomy, and the 12-month redislocation rate.

**Methods:**

This prospective cohort study included 40 non-ambulatory children with spastic cerebral palsy (50 hips; mean age 6.54 ± 1.07 years). Procedure selection was based on preoperative radiographic findings and intraoperative hip stability. Thirteen hips underwent VDRO alone, and 37 hips underwent VDRO combined with Dega pelvic osteotomy for acetabular dysplasia (acetabular index > 25°) or intraoperative posterolateral instability. Primary aim was to measure migration percentage and acetabular index preoperatively and during 12-months follow-up. Secondary aim was to assess 12-month redislocation rate and caregiver-reported quality of life using the Caregiver Priorities and Child Health Index of Life with Disabilities (CPCHILD) questionnaire at baseline and 12 months. Analyses used generalized estimating equation models (GEE) to account for bilateral hips and repeated measures.

**Results:**

In the overall cohort, migration percentage improved from 88.12 to 20.64 and acetabular index from 30.65 to 25.01 at 12 months (both *p* < 0.001). In exploratory subgroup analyses, 12-month migration percentage decreased from 70.83 to 33.15 after VDRO alone and from 93.94 to 15.99 after VDRO + Dega. Redislocation occurred in 8 of 50 hips (16.0%) overall, including 46.2% after VDRO alone and 5.4% after VDRO + Dega. CPCHILD total scores improved significantly in both groups, with no significant between-group difference in change.

**Conclusions:**

Reconstructive hip surgery was associated with short-term radiographic improvement and improved caregiver-reported outcomes in this cohort of non-ambulatory children with spastic cerebral palsy. Although 12-month redislocation was lower after VDRO + Dega, the non-randomized design, baseline imbalance, and short follow-up limit comparative inference.

## Background

Hip subluxation and dislocation are common musculoskeletal complications in children with cerebral palsy (CP), with an estimated prevalence of approximately 45% of hips [1]. The severity of motor damage is classified according to the Gross Motor Function Classification System (GMFCS), which relates with the risk of orthopaedic complications [2]. Children classified as GMFCS levels IV and V are non-ambulatory and highly dependent on caregivers for daily activities, placing them at particularly high risk of progressive hip displacement [3]. In this non-ambulatory group, the frequency of hip displacement may reach up to 90%, often progress to complete hip dislocation, which can lead to severe hip pain, an uneven pelvic alignment where one side of the pelvis is higher than the other, impaired sitting, difficulties with hygiene and care of perineal area, and a marked reduction in children’s overall well-being [4].

Proximal femoral varus derotational osteotomy (VDRO), frequently combined with a pelvic osteotomy such as the Dega procedure, is among the most frequently performed reconstructive surgeries for hip displacement in children with cerebral palsy [5].

The primary aim of reconstructive hip surgery is to achieve and maintain stable femoral head containment within the acetabulum, thereby reducing the risk of redislocation and restoring a centered, stable hip [6]. Traditionally, surgical success has been primarily evaluated based on radiographic outcomes, particularly through reduction or stabilization of the migration percentage (MP). However, in non-ambulatory children, real success also depends on improvements in well-being, ease of care, and overall daily activity, rather than radiographic correction alone [7].

The main functional aims of surgery therefore include alleviation of pain, improved sitting tolerance, and easing of caregiving, eventually enhancing quality of life for both the child and caregivers [8]. The Caregiver Priorities and Child Health Index of Life with Disabilities (CPCHILD) questionnaire was developed and validated to assess health-related quality of life in children with severe cerebral palsy, particularly those classified as GMFCS levels III to V [9]. Therefore, the purpose of this prospective study was to evaluate the outcomes of reconstructive hip surgery as VDRO alone and VDRO combined with Dega pelvic osteotomy in non-ambulatory children with spastic cerebral palsy concerning postoperative radiological stability and caregiver-reported functional outcomes.

## Methods

### Study design and participants

This prospective cohort study included non-ambulatory children with spastic cerebral palsy who presented with hip subluxation or dislocation between January 2022 and December 2024 at Mansoura University Hospitals, a tertiary referral centre. Children were excluded if they had a history of femoral or pelvic osteotomy on the affected side, windswept deformity, non-spastic forms of cerebral palsy, pressure ulcers around the hip region, or any medical condition that made them unfit for surgery. All participants underwent a comprehensive preoperative evaluation, including documentation of demographic characteristics (age and sex), Gross Motor Function Classification System (GMFCS) level, distribution of cerebral palsy, and relevant medical and surgical history.

### Ethics approval and consent to participate

The study was conducted in accordance with the Declaration of Helsinki and approved by the Institutional Review Board of Mansoura University Faculty of Medicine (Approval No. MD.22.8.679; August 24, 2022). Informed written consent was obtained from the parents or legal guardians of all participants before their enrollment in the study.

### Clinical trial number

Not applicable.

### Preoperative evaluation

Clinical evaluation included assessment of hip range of motion, particularly limited abduction, hip flexion contracture using the Thomas test, limb length discrepancy, and femoral rotational alignment. Radiological evaluation consisted of an anteroposterior pelvic radiograph obtained in a neutral position. The migration percentage (MP) and acetabular index (AI) were measured. In selected complex cases, computed tomography (CT) was performed to help in surgical planning for pelvic osteotomy.

### Surgical intervention

All procedures were performed using an extra-articular approach for reconstruction of hip displacement in children with cerebral palsy. Proximal femoral varus derotational and shortening osteotomy (VDRO) was performed in all hips, with or without a Dega transiliac pelvic osteotomy. Open reduction was not routinely required, as concentric reduction was achieved following soft-tissue release and VDRO. Soft-tissue release was initially performed through a transverse incision below the groin crease, including sequential release of the adductor longus, gracilis, and adductor brevis muscles. VDRO was then carried out at the level of the lesser trochanter. A trapezoidal bone wedge was resected, including the iliopsoas insertion, to achieve femoral shortening and varisation, aiming for a postoperative neck–shaft angle between 110° and 130°. The proximal fragment was abducted and internally rotated under fluoroscopic guidance until concentric femoroacetabular reduction was confirmed. Fixation was achieved using a locking plate. In hips demonstrating acetabular dysplasia (acetabular index > 25°) or intraoperative posterolateral instability detected based on intraoperative arthrography, dynamic radiography, and provocative examination after VDRO, a Dega pelvic osteotomy was additionally performed based on the senior surgeon’s assessment of hip stability, reduction, and femoral head coverage.

### Postoperative follow-up

Postoperatively, patients were immobilised in a hip spica cast for 4–6 weeks. Short-term follow-up evaluations were scheduled at 1–2 weeks, 4–6 weeks (for cast removal), and at 3, 6, and 12 months after surgery. Migration percentage (MP) and acetabular index (AI) were measured at each follow-up visit. Redislocation was defined as a migration percentage greater than 60% on follow-up radiographs, representing substantial loss of reduction and severe subluxation prior to complete dislocation. Although no universally accepted threshold exists, this definition has been widely used in previous studies [10, 11]. During the recovery phase, antispasmodic medications were continued to control spasticity and improve comfort. Physiotherapy was initiated immediately after cast removal to preserve hip range of motion and promote functional outcomes**.**

### Short-term outcome measures

Radiographic measures were the main outcome of this study for two subgroups of reconstructive hip surgery as VDRO alone and VDRO combined with Dega pelvic osteotomy in non-ambulatory children with spastic cerebral palsy and were determined by longitudinal changes in migration percentage (MP) and acetabular index (AI) on standard anteroposterior pelvic radiographs obtained at 3, 6 and 12 months following surgery. As a secondary outcome, rate of redislocation at the 12-months short-term follow-up was evaluated. The tertiary outcome was to evaluate postoperative changes in the overall CPCHILD score from baseline to 12-months, representing caregiver-reported improvements in health-related quality of life.

### Statistical analysis

All statistical analyses were performed using SPSS version 26 (IBM Corp., Armonk, NY, USA). Continuous variables are presented as mean ± standard deviation and categorical variables as number and percentage. To account for bilaterality and repeated measurements within the same child, generalized estimating equation (GEE) models with an exchangeable correlation structure were used. For continuous outcomes, including migration percentage (MP), acetabular index (AI), and CPCHILD scores, Gaussian GEE models were applied to estimate mean changes from baseline with 95% confidence intervals and *p* values. Between-group differences in change were assessed using the time × group interaction term. For 12-month redislocation, exploratory clustered logistic GEE models were used, and results are reported as odds ratios with 95% confidence intervals. A two-sided *p* value < 0.05 was considered statistically significant. Because procedure allocation was non-randomized and the number of redislocation events was small, results from the exploratory multivariable model were interpreted cautiously, and residual confounding by indication may remain.

## Results

### Baseline demographic and clinical characteristics

The study included 50 hips from 40 children with spastic cerebral palsy who underwent reconstructive hip surgery. The participants had a mean age of 6.54 ± 1.07 years (range: 5–9 years). Most children had severe motor impairment, with 67.5% (27 children) classified as GMFCS Level V and 32.5% (13 children) as Level IV. In terms of anatomical distribution, quadriplegia was the most common pattern (52.5%), followed by diplegia (40%) and hemiplegia (7.5%).

VDRO combined with Dega pelvic osteotomy was the reconstructive hip surgery performed in 74% of hips (37 hips), while the remaining 13 hips (26%) underwent VDRO alone. Preoperative radiographs revealed severe hip deformity throughout the study group, with a mean migration percentage (MP) of 87.9 ± 18.3, and 92% of hips demonstrated an MP higher than 50%. The mean preoperative acetabular index (AI) was 30.59 ± 5.90, indicating substantial acetabular dysplasia (Table 1).

**Table 1:** Baseline demographic and clinical characteristics of the study cohort

Variables	Study participants N (%) (*n* = 50 hips; 40 children)	
Age (years)	Mean ± SD	6.54 ± 1.07
	Range (years)	5–9
Sex	Female	22 (55%)
	Male	18 (45%)
Laterality	Unilateral	30 (75%)
	Bilateral	10 (25%)
Joint side	Right	23 (46%)
	Left	27 (54%)
GMFCS level	IV	13 (32.5%)
	V	27 (67.5%)
Comorbidity	None	24 (60%)
	Convulsions	15 (37.5%)
	Convulsions + Psoriasis	1 (2.5%)
Distribution	Hemiplegic	3 (7.5%)
	Diplegic	16 (40%)
	Quadriplegic	21 (52.5%)
Reconstructive procedure	VDRO + Adductor release	13 (26%)
	VDRO + DEGA + Adductor release	37 (74%)
Preoperative migration percentage	Mean ± SD	87.9 ± 18.3
	Range	46.0–100.0
	MP > 50%	46 (92%)
	MP < 50%	4 (8%)
Preoperative acetabular index	Mean ± SD	30.59 ± 5.90
	Range	23.5–45.0
Redislocation rate	N (%)	8 (16%)

Values are presented as mean ± SD, range, or number (%), as appropriate. Child-level variables were summarized for 40 children, whereas hip-level variables were summarized for 50 hips

*Abbreviations GMFCS* Gross Motor Function Classification System, *VDRO* Varus derotational osteotomy, *Dega* Dega pelvic osteotomy, *MP* Migration percentage, *AI* Acetabular index

**Fig. 1:**
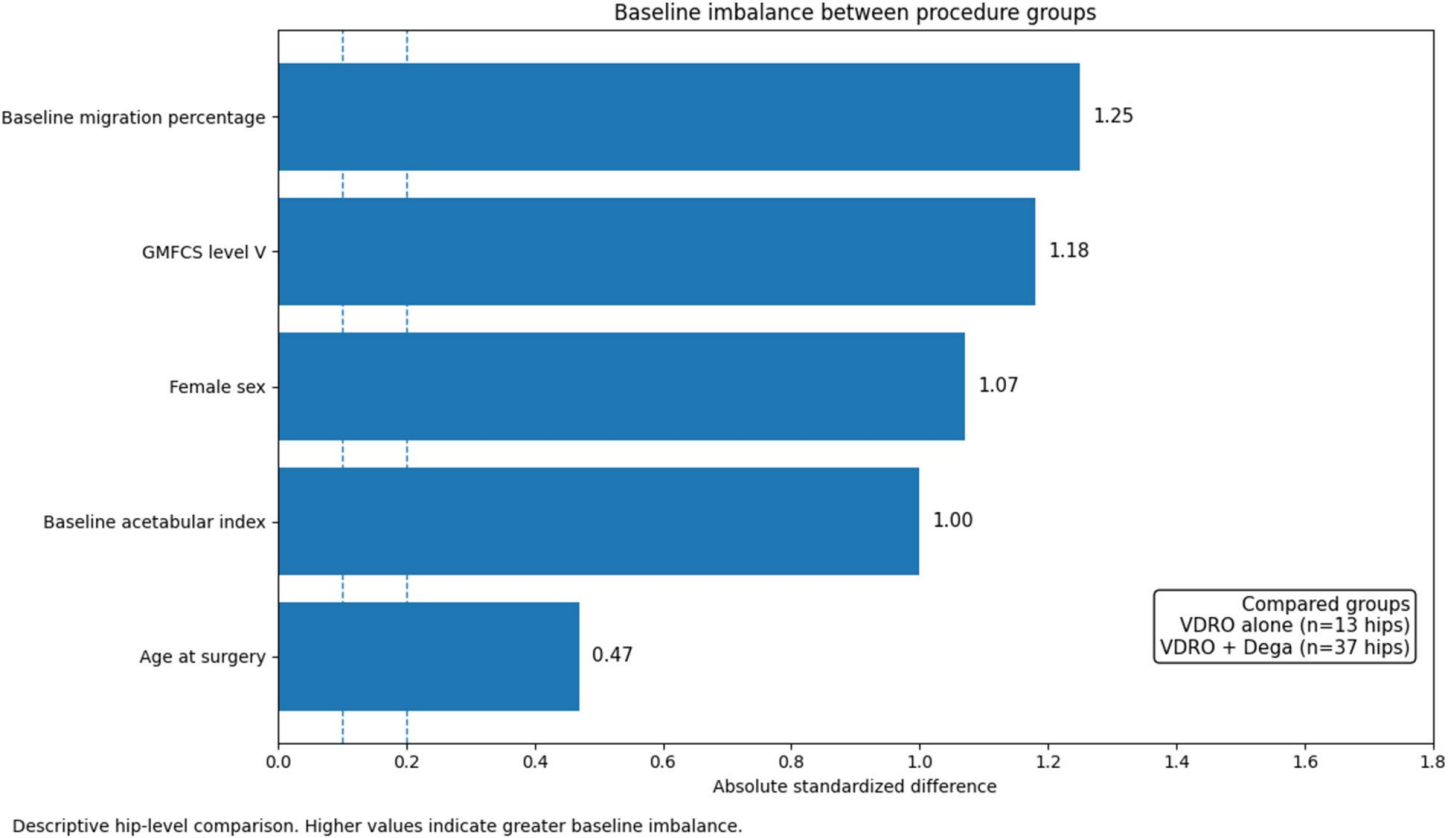
Differences in baseline characteristics between procedure subgroups

Table 2 shows clear baseline imbalance between procedure groups. Compared with hips treated with VDRO alone, hips treated with VDRO combined with Dega pelvic osteotomy were more often from children classified as GMFCS V and had greater preoperative radiographic severity, with higher baseline migration percentage and slightly higher acetabular index. Age and neurologic distribution were broadly similar between groups. Overall, these baseline differences suggest that the combined procedure was more frequently used in more severely involved hips, which should be considered when interpreting later between-group outcomes (Fig. 1).

**Table 2:** Baseline demographic, clinical, and radiographic characteristics by procedure group

Variable	VDRO alone (*n* = 13 hips)	VDRO + Dega (*n* = 37 hips)
Age at surgery (years), mean ± SD	6.19 ± 0.85	6.66 ± 1.12
Female sex, n (%)	3 (23.1%)	26 (70.3%)
GMFCS IV, n (%)	9 (69.2%)	7 (18.9%)
GMFCS V, n (%)	4 (30.8%)	30 (81.1%)
Quadriplegic distribution, n (%)	7 (53.8%)	20 (54.1%)
Diplegic distribution, n (%)	6 (46.2%)	14 (37.8%)
Hemiplegic distribution, n (%)	0 (0.0%)	3 (8.1%)
Baseline migration percentage, mean ± SD	70.83 ± 23.39	93.89 ± 11.46
Baseline acetabular index (°), mean ± SD	27.10 ± 2.21	31.81 ± 6.31

Values are presented as mean ± SD or n (%), as appropriate. Procedure groups were defined at the hip level. Therefore, for children contributing bilateral hips or different procedures in each hip, child-level characteristics (for example age, sex, GMFCS level, and distribution) could contribute to more than one procedure group. Accordingly, this table is intended to describe baseline imbalance between procedure groups rather than to support formal between-group comparison

*Abbreviations VDRO* Varus derotational osteotomy, *GMFCS* Gross Motor Function Classification System, *SD* Standard deviation

### Radiological outcomes

In the overall cohort, both radiographic measures showed significant improvement from baseline across all postoperative time points. MP decreased from an estimated baseline mean of 88.12 to 2.46 at 3 months, 10.62 at 6 months, and 20.64 at 12 months, with significant mean reductions from baseline at each time point (all *p* < 0.001). Similarly, AI improved from an estimated baseline mean of 30.65 to 25.32 at 3 and 25.18 at 6 months and 25.01 at 12 months, with all changes from baseline remaining statistically significant (all *p* < 0.001) (Table 3).

**Table 3:** Radiographic outcomes over short-term follow-up in the overall cohort

Radiographic measure	Baseline estimated mean	Time point	Estimated mean at time point	Estimated mean change from baseline (β)	95% CI	*P*-value
MP	88.12	3 months	2.46	−85.66	−91.33 to −79.99	< 0.001
		6 months	10.62	−77.50	−83.75 to −71.25	< 0.001
		12 months	20.64	−67.48	−74.95 to −60.01	< 0.001
AI	30.65	3 months	25.32	−5.34	−6.30 to −4.38	< 0.001
		6 months	25.18	−5.48	−6.45 to −4.50	< 0.001
		12 months	25.01	−5.64	−6.81 to −4.48	< 0.001

Values are presented as model-estimated means and mean changes from baseline (β) with 95% confidence intervals. Baseline was the reference time point. Estimates were obtained using GEE models accounting for repeated measures and within-child correlation. Negative β values indicate a reduction from baseline

*Abbreviations MP* Migration percentage, *AI* Acetabular index, *CI* Confidence interval

**Fig. 2:**
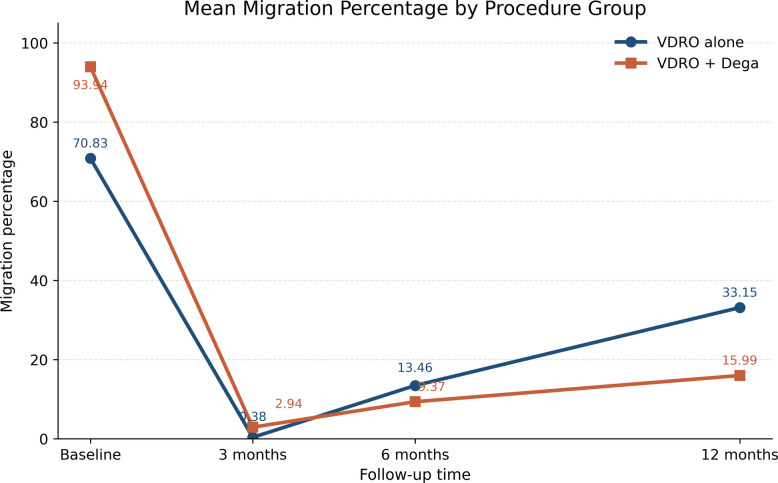
Mean migration percentage over short-term follow-up by procedure subgroup

**Fig. 3:**
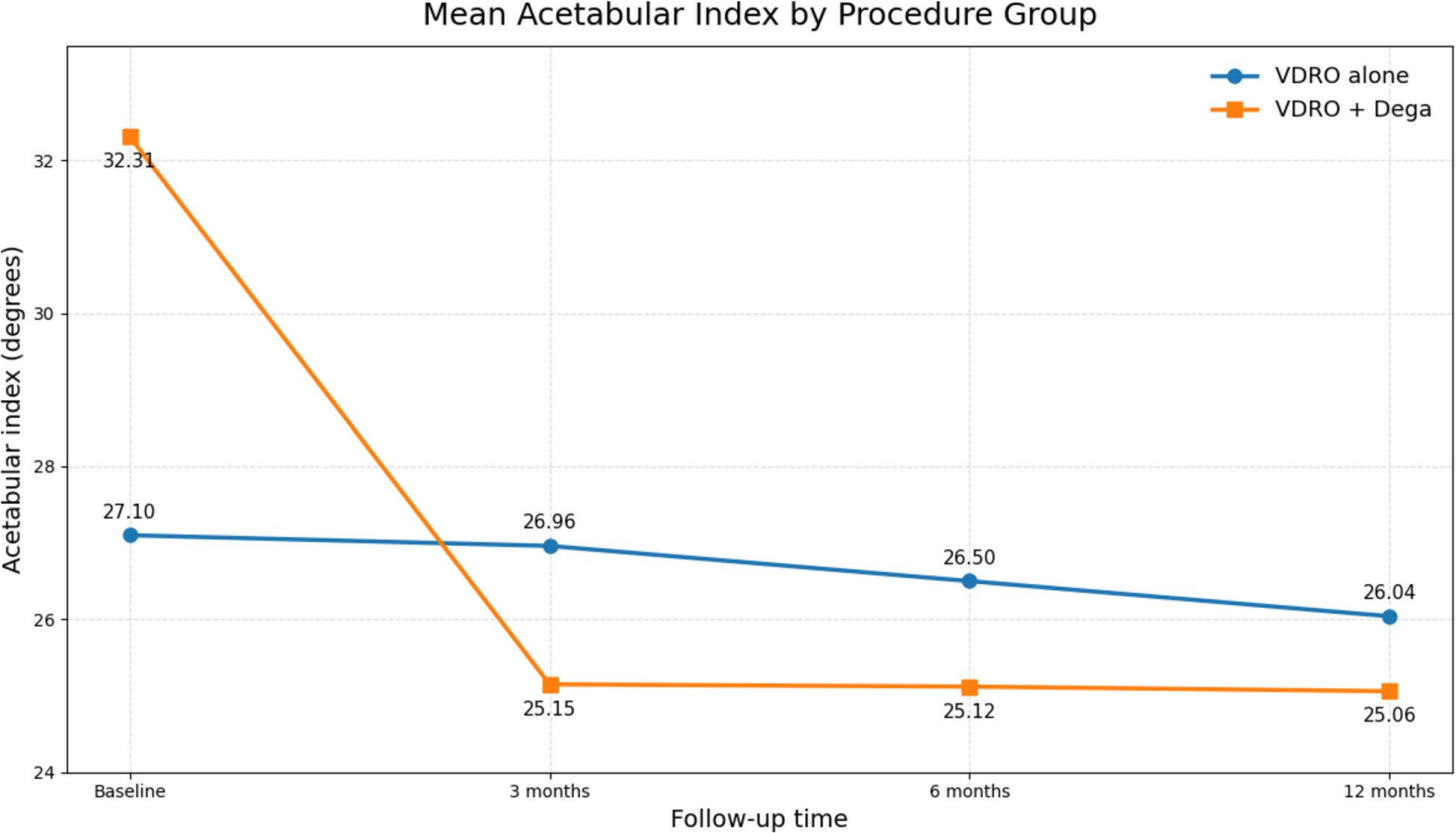
Mean acetabular index over short-term follow-up by procedure subgroup

Radiographic improvement was observed over short-term follow-up in both surgical subgroups. In the VDRO alone group, estimated mean MP declined from 70.83 at baseline to 0.38, 13.46, and 33.15 at 3, 6, and 12 months, respectively, while estimated mean AI declined from 27.10 to 26.96, 26.50, and 26.04. In the VDRO + Dega group, estimated mean MP declined from 93.94 at baseline to 2.94, 9.37, and 15.99, and estimated mean AI declined from 32.31 to 25.15, 25.12, and 25.06 at the same short-term follow-up points. Within-group changes from baseline remained statistically significant across all postoperative assessments for both MP and AI (all *p* < 0.05) (Table 4) (Figs. 2 and 3).

**Table 4:** Radiographic outcomes over short-term follow-up for subgroup procedure

Variable	Time point	VDRO alone			VDRO + Dega		
		**Baseline** **mean**	**Estimated** **mean**	**β (95% CI), p**	**Baseline** **mean**	**Estimated** **mean**	**β (95% CI), p**
**MP**	3 months	70.83	0.38	−70.45 (−82.88 to −58.01); < 0.001	93.94	2.94	−91.00 (−95.24 to −86.77); < 0.001
	6 months		13.46	−57.37 (−66.77 to −47.97); < 0.001		9.37	−84.57 (−89.74 to −79.40); < 0.001
	12 months		33.15	−37.68 (−47.78 to −27.57); < 0.001		15.99	−77.95 (−83.20 to −72.70); < 0.001
**AI**	3 months	27.10	26.96	−0.14 (−0.40 to 0.12); 0.298	32.31	25.15	−7.16 (−7.92 to −6.41); < 0.001
	6 months		26.50	−0.60 (−0.99 to −0.21); 0.002		25.12	−7.19 (−8.01 to −6.37); < 0.001
	12 months		26.04	−1.06 (−1.67 to −0.45); 0.001		25.06	−7.25 (−8.50 to −6.00); < 0.001

Values are GEE-estimated means from separate models within each procedure group, using baseline as the reference time point. β denotes the estimated mean change from baseline with 95% confidence intervals and *p* values. Models accounted for bilaterality by clustering on case number

*Abbreviations VDRO* Varus derotational osteotomy, *MP* Migration percentage, *AI* Acetabular index, *CI* Confidence interval

### Redislocation Rate

**Fig. 4:**
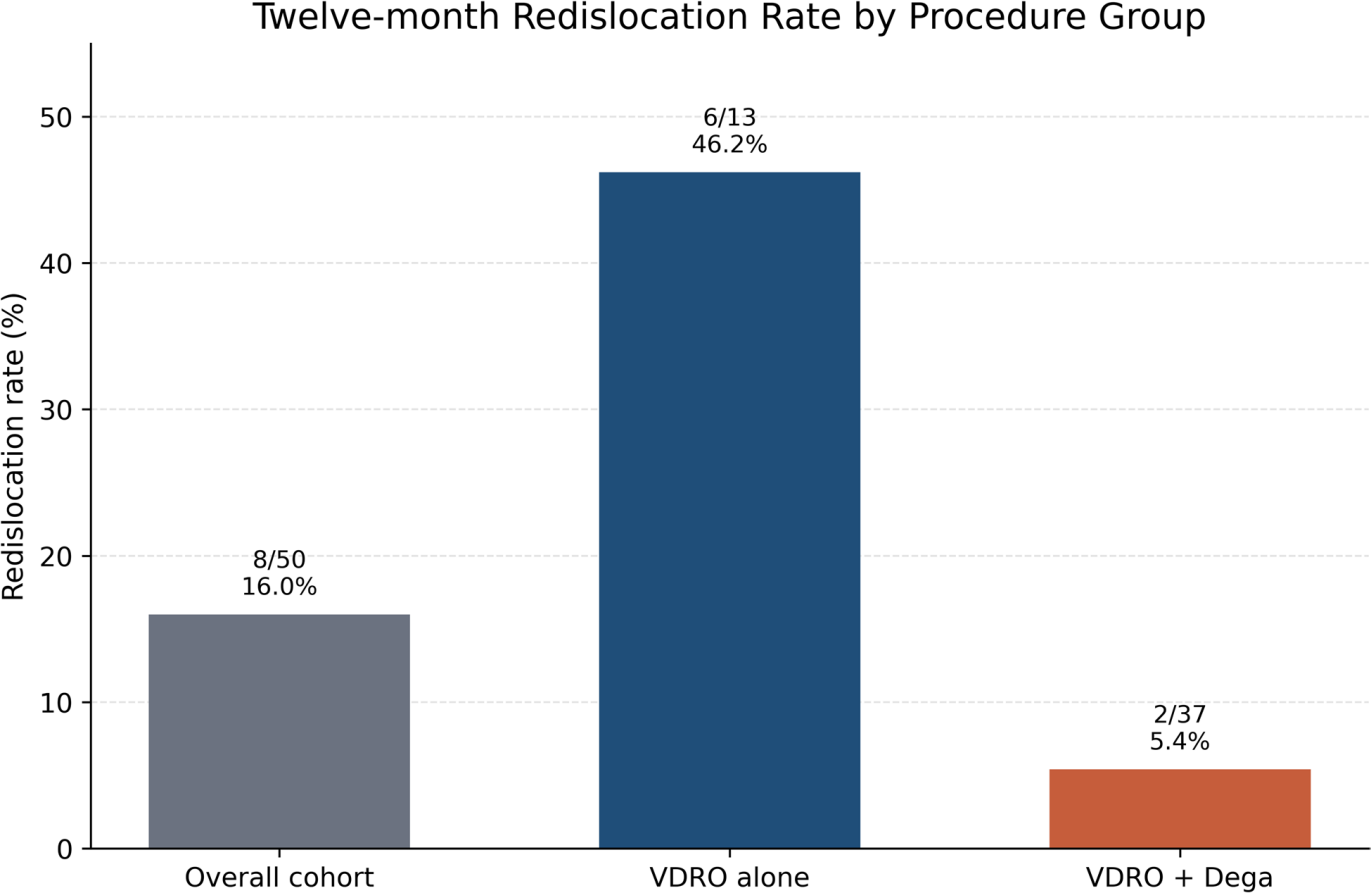
Redislocation rates at 12-month follow-up by procedure subgroup

**Fig. 5:**
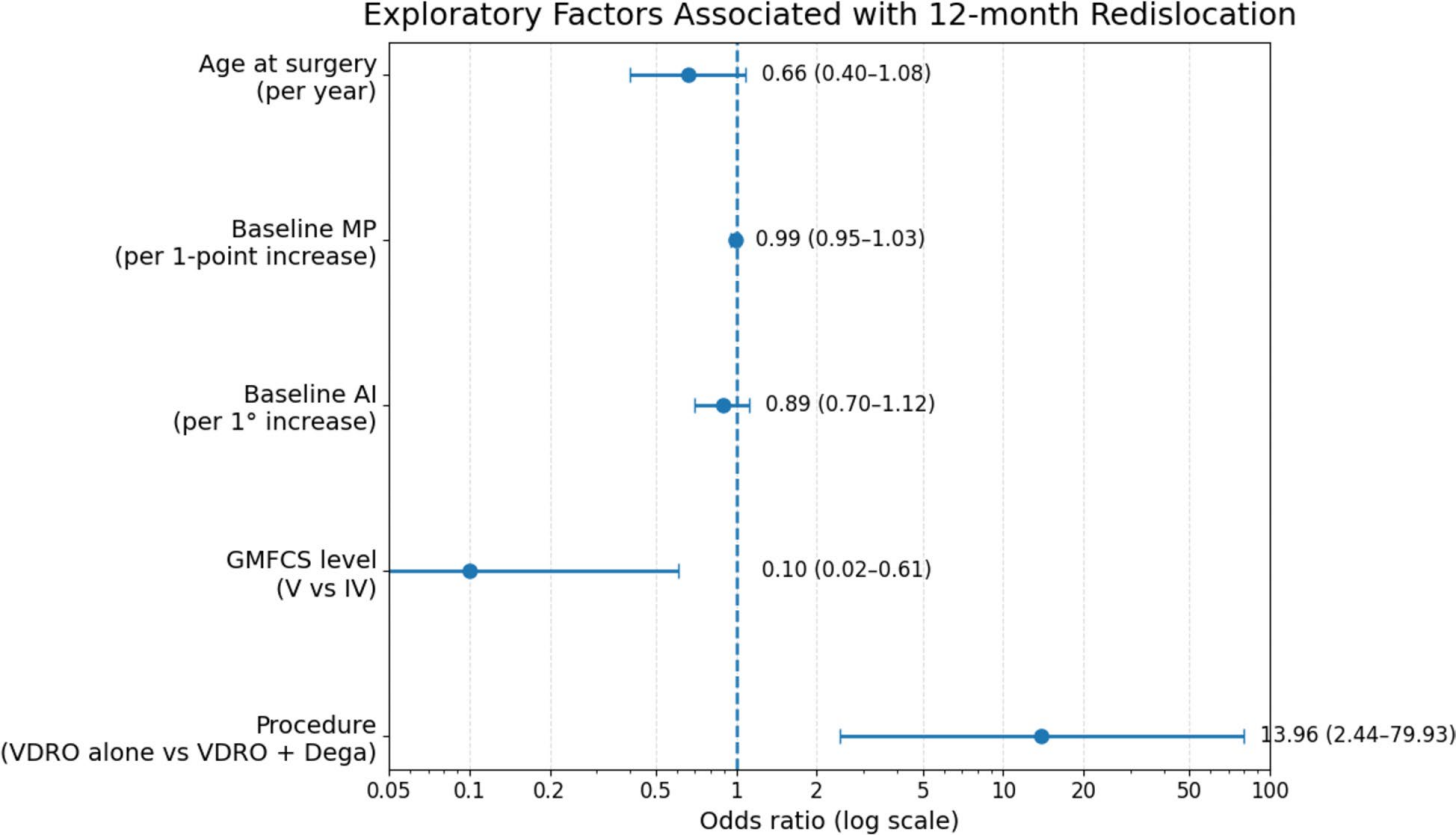
Exploratory factors associated with 12-month redislocation rate

At 12 months, redislocation was observed in 8 of 50 hips (16.0%). In exploratory clustered analysis, GMFCS level V was associated with lower odds of redislocation compared with level IV (OR 0.10, 95% CI 0.02 to 0.61; *p* = 0.013), whereas VDRO alone was associated with higher odds of redislocation than VDRO + Dega (OR 13.96, 95% CI 2.44 to 79.93; *p* = 0.003). Given the small number of events, the limited 12-month short-term follow-up, and baseline differences between procedure groups, these findings should be interpreted with caution as exploratory associations and not as definitive evidence of comparative treatment effect (Table 5) (Figs. 4 and 5).

**Table 5:** Twelve-month redislocation rate by procedure group and exploratory analysis of factors associated with 12-month redislocation

Procedure group	Redislocation rate, N (%)		
Overall cohort	8/50 (16.0%)		
VDRO alone	6/13 (46.2%)		
VDRO + Dega	2/37 (5.4%)		
Exploratory analysis of factors associated with 12-month redislocation rate			
Variable	Odds ratio (OR)	95% CI	*P*
Age at surgery (per year)	0.66	0.40 to 1.08	0.097
Baseline MP (per 1% increase)	0.99	0.95 to 1.03	0.605
Baseline AI (per 1° increase)	0.89	0.70 to 1.12	0.309
GMFCS level (V vs IV)	0.10	0.02 to 0.61	0.013
Procedure (VDRO alone vs VDRO + Dega)	13.96	2.44 to 79.93	0.003

For procedure type, an odds ratio greater than 1 indicates higher odds of redislocation in the VDRO-alone group relative to the VDRO + Dega group. For GMFCS, the comparison reflects level V versus level IV

*VDRO* Varus derotational osteotomy, *Dega* Dega pelvic osteotomy, *MP* Migration percentage, *AI* Acetabular index, *GMFCS* Gross Motor Function Classification System, *OR* Odds ratio, *CI* Confidence interval

### CPCHILD scores for clinical short-term follow-up

Table 6 shows total CPCHILD scores improvement during short-term follow-up from baseline to 12-months in both surgical groups from 40.85 to 48.60 in the VDRO alone group and from 35.98 to 44.53 in the VDRO + Dega group. The between-group difference in change was not statistically significant (βint = 0.80, 95% CI −1.30 to 2.90; *p* = 0.457).

**Table 6:** Short-term 12-months follow-up of CPCHILD subscale and total scores, with estimated within-group change and between-group differences by procedure group

Subscale	VDRO alone			VDRO + Dega			Between-group Δ change βint (95% CI), *p*
	**Preoperative**	**Postoperative** **12 months**	**β (95% CI), *p***	**Preoperative**	**Postoperative** **12 months**	**β (95% CI), *p***	
Activities of daily living	45.03	57.79	12.76 (8.72 to 16.81); < 0.001	43.07	51.88	8.81 (6.81 to 10.82); < 0.001	−3.95 (−7.83 to −0.08); 0.046
Positioning, transfer and mobility	31.27	40.88	9.62 (7.39 to 11.84); < 0.001	27.56	35.92	8.36 (6.07 to 10.65); < 0.001	−1.25 (−4.44 to 1.93); 0.441
Comfort and emotion	41.54	50.96	9.42 (6.47 to 12.38); < 0.001	36.79	48.57	11.78 (8.80 to 14.76); < 0.001	2.36 (−2.10 to 6.81); 0.300
Communication and social interaction	48.39	51.04	2.65 (1.21 to 4.08); < 0.001	45.94	49.30	3.35 (1.42 to 5.28); < 0.001	0.71 (−1.70 to 3.11); 0.565
Health	42.08	51.04	8.96 (7.03 to 10.89); < 0.001	40.20	48.58	8.38 (7.49 to 9.28); < 0.001	−0.58 (−2.70 to 1.55); 0.594
Quality of life	31.61	43.46	11.85 (9.13 to 14.58); < 0.001	25.33	33.19	7.86 (6.09 to 9.64); < 0.001	−3.99 (−7.12 to −0.85); 0.013
Total CPCHILD score	40.85	48.60	7.75 (6.31 to 9.19); < 0.001	35.98	44.53	8.54 (6.95 to 10.14); <0.001	0.80 (−1.30 to 2.90); 0.457

Values are presented as estimated means. Within-group β represents the estimated change from preoperative to 12 months, and between-group β represents the estimated difference in change between groups

*CI* Confidence interval, *VDRO* Varus derotational osteotomy, *CPCHILD* Caregiver Priorities and Child Health Index of Life with Disabilities. Analyses accounted for bilaterality

**Fig. 6:**
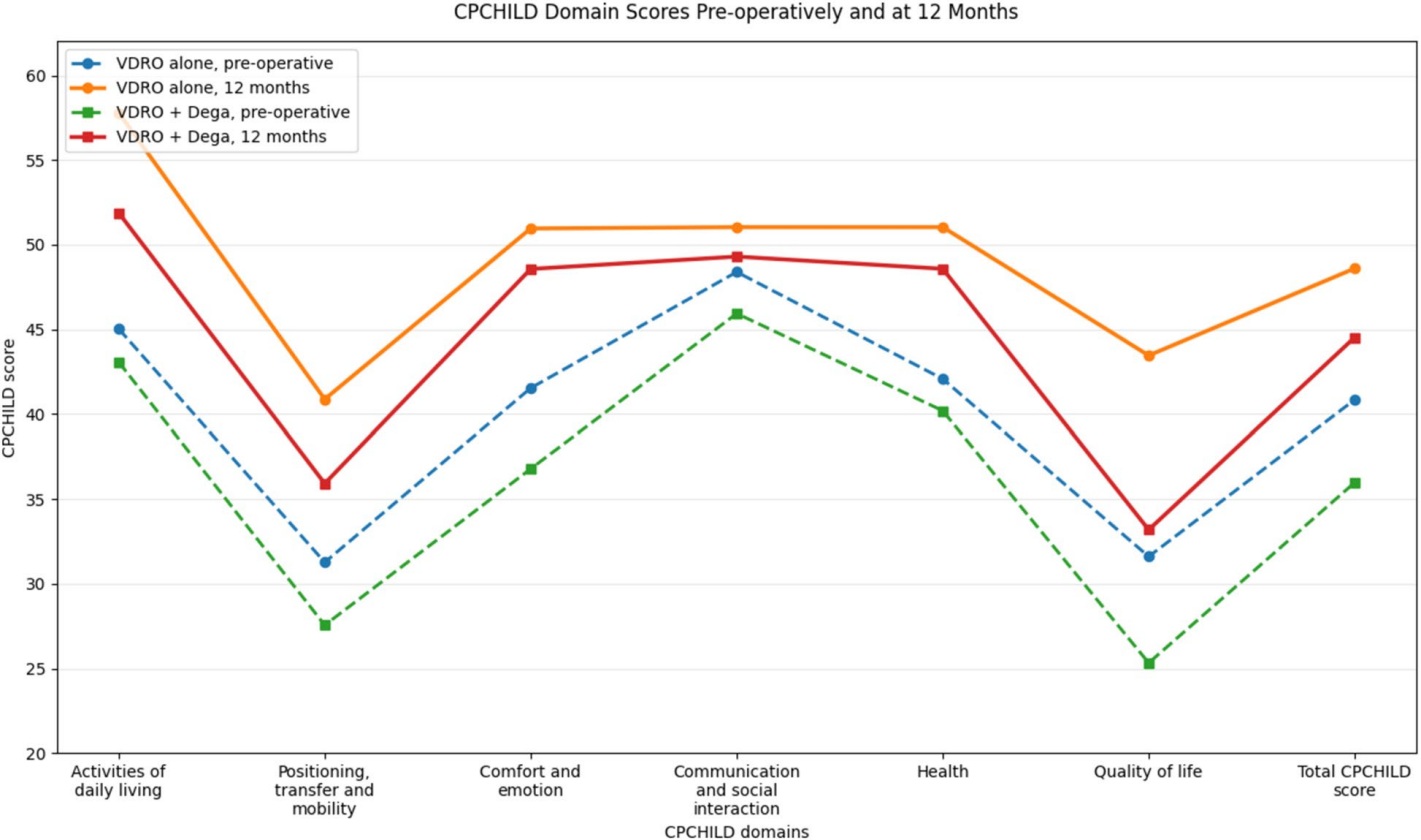
Total CPCHILD score and subscales before surgery and at 12-months according to procedure subgroup

Full domain-level CPCHILD results are presented in Table 6 as both procedure groups demonstrated significant improvement from baseline to 12 months across all CPCHILD subscales (all within-group *p* < 0.001). Exploratory between-group analyses showed no significant differences in the magnitude of change for most domains or for the total score, Given the baseline imbalance between groups and the non-randomized treatment allocation, these between-group findings should be interpreted cautiously and considered exploratory (Fig. 6).

## Discussion

Hip instability and progressive displacement remain major challenges in non-ambulatory children with spastic CP, particularly in those classified as GMFCS levels IV and V [3, 4]. Reconstructive surgery aims not only to restore radiographic containment but also to improve comfort, ease of care, and overall quality of life [6, 8]. The present study assessed radiological stability and caregiver-reported clinical outcomes following one-stage hip reconstruction and results of subgroup procedures as VDRO alone and VDRO combined with Dega pelvic osteotomy.

Neither our study nor the available comparative literature used randomized treatment allocation [6, 11–17]. Most published data were derived from observational cohorts or case series, and even pooled estimates were based on non-randomized studies. Moreover, only a limited number of reports directly compared VDRO alone with combined femoral-pelvic reconstruction, most notably Axt and Wadley [15], Huh et al. [16], and the pooled analysis by Delbrück et al. [11], whereas several other studies described overall reconstructive outcomes without formal head-to-head comparison [6, 12–14, 17]. Methodologically, our study used GEE to account for bilaterality and repeated measurements, aligning with Axt and Wadley [15] and differing from the conventional tests, ANOVA-based methods, mixed-model approaches, or non-longitudinal analyses used in several other reports [6, 10–13, 16]. Our series included 50 hips in 40 children, compared with 74 hips in 57 children in Axt and Wadley [15], 116 hips in 75 children in Huh et al. [16], 141 hips in 95 patients in Sarıkaya et al. [12], and 168 hips in 121 patients in Rutz et al. [14], and was much smaller than the pooled dataset of 1540 hips in Delbrück et al. [11], although it was larger than Mubarak et al. [17] (18 hips) and slightly larger than DiFazio et al. [6] (38 patients). Our cohort was also modest in size, particularly in the VDRO-alone subgroup, which limits the precision of between-procedure estimates and reduces power for exploratory comparative analyses.

Follow-up in our cohort was limited to 12 months, whereas Huh et al. [16] reported 4.6 years, Sarıkaya et al. [12] 5 years, Mubarak et al. [17] 6 years and 10 months, Schörle and Manolikakis [13] and Rutz et al. [14] 7.3 years, and DiFazio et al. [6] 24 months. Taken together, these features indicate that our between-procedure findings are best regarded as short-term observational comparisons rather than definitive evidence of longer-term comparative effectiveness.

In our cohort, the decision to add Dega osteotomy was based on acetabular dysplasia and intraoperative posterolateral instability, broadly consistent with studies that used acetabular deficiency and intraoperative stability to guide extension of reconstruction [12, 13, 16, 17]. Wang et al. [10] further supports the anatomical basis for this approach by showing that acetabular dysplasia becomes more pronounced with increasing migration percentage, particularly at higher MP values. Shea et al. reported that posterolateral instability was considered present when, after VDRO, the hip remained unstable on intraoperative dynamic assessment, with failure to maintain stable concentric reduction and satisfactory femoral head containment, as judged by arthrography, dynamic radiography, and provocative examination [18]. However, indications were not uniform across the literature, as some reports relied more on surgeon preference, child health status, or broader reconstructive protocols rather than a directly comparable operative rule [11, 14, 15].

Redislocation was observed in 8 of 50 hips (16.0%) at 12 months in our cohort, including 6 of 13 hips (46.2%) after VDRO alone and 2 of 37 hips (5.4%) after VDRO + Dega. This overall rate was identical to the pooled redisplacement estimate reported by Delbrück et al. [11] (16%; 95% CI 12–21%) and broadly comparable to the 18% redislocation reported by Huh et al. [16] in a dislocated subgroup after combined reconstruction. In contrast, Sarıkaya et al. [12] reported an overall failure rate of 5.6%−5.7% (8 of 141 hips), DiFazio et al. [6] reported no revision surgery during 2-year follow-up, Schörle and Manolikakis [13] reported no reluxation in their reconstructive subgroup, and Mubarak et al. [17] described durable maintenance of reduction in 17 of 18 hips after combined one-stage reconstruction. In our exploratory model, redislocation was associated with procedure type and GMFCS level, with higher odds after VDRO alone than after VDRO + Dega (OR 13.96, 95% CI 2.44–79.93; *p* = 0.003) and lower odds in GMFCS V than GMFCS IV (OR 0.10, 95% CI 0.02–0.61; *p* = 0.013), whereas age (OR 0.66, *p* = 0.097), baseline MP (OR 0.99, *p* = 0.605), and baseline AI (OR 0.89, *p* = 0.309) were not significant. This partly aligns with Delbrück et al. [11], who also identified type of bony surgery as an important determinant of redislocation, but differs from Sarıkaya et al. [12], where unilateral surgery appeared more important and GMFCS, age, preoperative migration index, medial capsulotomy, and Dega osteotomy were not identified as risk factors. Because our cohort was limited to non-ambulatory children at GMFCS IV-V, whereas some studies included a wider functional spectrum or used different severity classifications [10, 12, 13], these comparisons should be interpreted in the context of differences in case mix, outcome definition, and follow-up.

Migration percentage improved after reconstruction in our cohort. In the overall cohort, MP decreased from an estimated baseline mean of 88.12 to 2.46 at 3 months, 10.62 at 6 months, and 20.64 at 12 months. In subgroup analysis, 12-month MP remained lower in the VDRO + Dega group than in the VDRO-alone group (15.99 vs 33.15), despite higher baseline MP in the combined group (93.94 vs 70.83). This pattern is consistent with Axt and Wadley [15], who likewise reported lower final MP after combined treatment than after VDRO alone (19.3% vs 35.7%). Broad agreement was also seen with Huh et al. [16], who reported final mean MP values of 26% and 28% in their two subgroups, Sarıkaya et al. [12], who reported improvement in migration index from 58.4 preoperatively to 10.0 postoperatively and 17.3 at final follow-up, DiFazio et al. [6], who reported a median migration percentage of 11% at 2 years, Schörle and Manolikakis [13], who reported reduction in Reimers migration index from 68 preoperatively to 24 at 6 months and 25 at 3–5 years, and Rutz et al. [14], who reported improvement from 77% preoperatively to 10% at final follow-up. Wang et al. [10] and Delbrück et al. [11] likewise support the importance of MP as a key marker of radiographic severity and outcome.

The increase in MP in the VDRO-alone group from 3 to 12 months likely reflects partial loss of containment over time after femoral correction without concurrent acetabular reconstruction. This interpretation is supported by Axt and Wadley [15], who showed poorer midterm stability after isolated VDRO than after VDRO combined with Dega osteotomy, and noted that the acetabulum is often dysplastic and not adequately addressed by femoral correction alone. Wang et al. [10], further showed that increasing migration is associated with progressive posterolateral acetabular dysplasia, supporting the idea that residual acetabular deficiency may contribute to recurrent lateral migration. In addition, Schörle and Manolikakis [13] emphasized that persistent muscular imbalance and abnormal joint biomechanics can continue to drive progressive decentration and eventual posterolateral/posterosuperior displacement. In our cohort, this pattern should still be interpreted cautiously because treatment allocation was non-random, the VDRO-alone subgroup was small, and follow-up was limited to 12 months. Acetabular index also improved over short-term follow-up. In the overall cohort, AI improved from an estimated baseline mean of 30.65 to 25.32 at 3 months and 25.18 at 6 months and 25.01 at 12 months. In subgroup analysis, AI improved from 27.10 to 26.04 in the VDRO-alone group and from 32.31 to 25.06 in the VDRO + Dega group at 12 months, with numerically greater within-group correction in the combined group. This is in keeping with Axt and Wadley [15], who reported better final AI after VDRO + Dega than after VDRO alone (16.9° vs 21.6°), Huh et al. [16], who reported final mean AI values of 25° and 29°, and Sarıkaya et al. [12], who reported improvement from 29.6 preoperatively to 24.7 postoperatively and 23.7 at final follow-up. Wang et al. [10] also provides anatomical support for the relationship between hip displacement and acetabular dysplasia. By contrast, DiFazio et al. [6] did not find a correlation between acetabular index and CPCHILD score, suggesting that radiographic acetabular correction may not fully parallel caregiver-perceived benefit.

In addition to radiographic improvement, our cohort showed significant postoperative clinical improvement. Total CPCHILD score increased from 40.85 to 48.60 in the VDRO-alone group and from 35.98 to 44.53 in the VDRO + Dega group, with estimated changes of 7.75 (95% CI 6.31–9.19; *p* = 0.001) and 8.54 (95% CI 6.95–10.14; *p* = 0.001), respectively, while the between-group difference in change was not significant (βint 0.80, 95% CI −1.30 to 2.90; *p* = 0.457). This extends the largely radiographic literature [11–13, 15, 16] by showing that reconstruction in our series was also associated with caregiver-reported functional benefit. Our findings are broadly consistent with DiFazio et al. [6], who reported an increase in mean total CPCHILD score from 49.6 at baseline to 58.9 at 12 and 24 months after surgery, and with Rutz et al. [14], who reported substantial reductions in pain intensity and pain frequency together with improved hip classification scores. Overall, our results support short-term radiographic and clinical improvement after reconstructive hip surgery in non-ambulatory children with spastic cerebral palsy, while between-procedure differences should still be considered in light of the observational design, baseline imbalance, modest sample size, and limited follow-up.

In this prospective cohort, reconstructive hip surgery was associated with short-term improvement in hip containment and caregiver-reported outcomes at 12 months in non-ambulatory children with severe spastic cerebral palsy.

### Limitations

This study has several limitations. Treatment allocation was non-randomized, and baseline severity differed between groups, so residual confounding by indication may remain despite the use of GEE models to account for bilaterality and repeated measurements. No a priori sample size calculation was performed because the cohort represented the eligible cases treated during the study period. Accordingly, the sample size was modest, particularly in the VDRO-alone subgroup, which limits the precision of between-group estimates. Follow-up was limited to 12 months, which restricts assessment of long-term durability and recurrence. In addition, the small number of redislocation events limits interpretation of the exploratory failure analysis. A standardized prospective assessment of perioperative medical complications was not available, and this limits interpretation of safety outcomes.

## Conclusions

Reconstructive hip surgery was associated with significant short-term radiographic improvement and improved caregiver-reported outcomes in non-ambulatory children with severe spastic cerebral palsy. Although lower 12-month redislocation rate was observed in hips treated with VDRO combined with Dega pelvic osteotomy, the non-randomized design and baseline imbalance between groups preclude causal inference regarding comparative effectiveness. Longer-term studies are needed to assess durability and clarify procedure-specific outcomes.

## Data Availability

The datasets generated and analyzed during the current study are available from the corresponding author on reasonable request.

## Abbreviations

AI: Acetabular index
CI: Confidence interval
CP: Cerebral palsy
CPCHILD: Caregiver Priorities and Child Health Index of Life with Disabilities
GMFCS: Gross Motor Function Classification System
MP: Migration percentage
VDRO: Varus derotational osteotomy
